# Stressors-induced cognitive dysfunction during aging: mechanisms and future challenges

**DOI:** 10.3389/fnagi.2025.1630982

**Published:** 2025-10-22

**Authors:** Yizhe Zhang, Xiao Zhang, Jiaxi Guo, Wanbing Dai, Sifan Chen, Lili Huang, Xuwu Xiang, Weifeng Yu, Diansan Su

**Affiliations:** ^1^Department of Anesthesiology, Renji Hospital, Shanghai Jiaotong University School of Medicine, Shanghai, China; ^2^Key Laboratory of Anesthesiology (Shanghai Jiaotong University), Ministry of Education, Shanghai, China; ^3^Department of Anesthesiology, The First Affiliated Hospital, Zhejiang University School of Medicine, Hangzhou, China; ^4^Department of Radiology, First Affiliated Hospital of Chongqing Medical University, Chongqing, China

**Keywords:** stress, cognitive dysfunctions, aging, ER stress, mitochondria

## Abstract

Stressful events can lead to transient impairments in learning and memory, a phenomenon more pronounced in the elderly. As global life expectancy rises, the shift toward an aging society underscores the urgent need for effective preventive strategies against stress-induced cognitive dysfunction. Elucidating its pathogenesis is essential for developing neuroprotective interventions and mitigating medical and societal impacts. In this study, male C57BL/6 mice aged 2 and 18 months were subjected to restraint stress (2 h/day for 14 days). Spontaneous activity and anxiety-like behavior were evaluated using the open field test, and cognitive performance was assessed via the novel object recognition test. mRNA sequencing revealed differentially expressed genes, which were further analyzed using Gene Ontology enrichment through the Database for Annotation, Visualization and Integrated Discovery (DAVID) database. Key molecular findings were validated by Quantitative Polymerase Chain Reaction (RT-qPCR), Western blot, and immunofluorescence. Additionally, a literature review was conducted to identify emerging research directions. Our results reveal that aged mice exhibit impaired upregulation of protective Endoplasmic Reticulum (ER) stress genes and show downregulation of mitochondrial expression and translation pathways, in contrast to young mice in which stress primarily upregulated genes involved in mitochondrial organization and Adenosine Triphosphate (ATP) metabolism. These age-specific vulnerabilities highlight ER stress and mitochondrial dysfunction as potential intervention targets.

## 1 Introduction

Cognitive decline is particularly pronounced in older individuals exposed to acute stress disorder (ASD), post-traumatic stress disorder (PTSD), and other major life stressors (Sapolsky, 1999; Biltz et al., 2022). With a rapidly aging global population, the burden of stress-related cognitive dysfunction has become a growing public health and socioeconomic concern (Nestler and Russo, 2024). Over the past few decades, a wealth of research has deepened our understanding of the physiological and pathophysiological interplay between stress and memory (Cameron and Glover, 2015; Jiang et al., 2019). Stress activates the body’s innate response systems—including the hypothalamic–pituitary–adrenal (HPA) axis and immune pathways—triggering complex molecular and cellular changes (Tomiyama, 2019; Steptoe and Kivimaki, 2012; McEwen, 2007). However, the age-associated mechanisms that exacerbate vulnerability to stress-induced cognitive dysfunction remain insufficiently characterized.

In this study, transcriptomic analysis revealed that aging-related cognitive dysfunction induced by stress is associated with alterations in pathways involved in synaptic signaling, neurogenesis, endoplasmic reticulum (ER) stress, mitochondrial function, amyloid metabolism, and lipoprotein regulation. While transcriptome sequencing provides high-resolution insights into molecular changes, it alone does not capture broader research trends or help prioritize future directions. To address this, we integrated transcriptomic findings with bibliometric analysis to identify both biologically relevant mechanisms and emerging research priorities.

Our findings highlight key molecular and thematic research fronts in the study of stress-induced cognitive decline during aging. Future investigations may benefit from a focus on organelle stress responses—particularly those involving the ER and mitochondria—as well as on lipid metabolism and amyloid processing pathways.

## 2 Materials and methods

### 2.1 Mice

This study received approval from the Animal Care and Use Committee of RenJi Hospital, Shanghai JiaoTong University School of Medicine, Shanghai, China (Approval Number: RJ2022-0918). All experimental procedures adhered to the guidelines outlined in the *Guide for the Care and Use of Laboratory Animals* by the National Institutes of Health and the U. K. *Animals (Scientific Procedures) Act*. Measures were taken to minimize distress and limit the number of animals used. This research was conducted following the ARRIVE guidelines (Percie du Sert et al., 2020). 18–20 months C57BL/6 J mice were obtained from the Shanghai Institute of Planned Parenthood Research-BK Laboratory Animal Limited Company (SIPPR-BK, China). They were kept in conventional cages under regulated experimental conditions (22 ± 2 °C, 12-h light/dark cycle) with free access to food and water. They were randomly assigned to experimental groups and housed in separate cages to prevent interactions that might influence treatment-dependent outcomes.

### 2.2 Restrain stress model

Restraint stress was carried out as described previously (Jiang et al., 2019; Snyder et al., 2011). Briefly, mice were confined to ventilated 50 mL conical tubes for 2 h daily. The tubes were perforated to ensure adequate airflow and unrestricted breathing. For the restraint stress (RS) model, the stress protocol was conducted from 9:00 a.m. to 11:00 a.m. each day and continued for a total of 14 days.

### 2.3 Behavioral tests

All behavioral tests were conducted during the light period between 9 a.m. and 6 p.m., and the apparatus was carefully cleaned by wiping with 70% ethanol after each trial to remove olfactory cues. All mice were familiarized with the researchers and habituated to the training room prior to the experiment. The animals to be tested were moved into the behavioral testing room 30 min before the start of the trial to minimize olfactory cues. The bedding of the animals’ home cages was not changed during the period of the behavioral tests. To minimize the variation introduced by experimenter handling, the mice were handled by the same experimenter throughout all behavioral experiments.

#### 2.3.1 Open field test (OFT)

Spontaneous activity and anxiety-like behaviors in the open field were assessed using an opaque plastic cube box (40 × 40 × 40 cm) under a camera. The center region was defined as a 15 cm × 15 cm area. To reduce anxiety in the animals, the light intensity in the center of the box was set to 100 ± 5 lux. Each mouse was first placed in the center of the box and given 15 min to move freely. The locomotor activity, mean speed, and time spent in the center were recorded by an overhead video camera and automatically analyzed using the ANY-maze behavioral tracking software (version 7.33, Stoelting Co., USA).

#### 2.3.2 Novel object recognition (NOR)

The NOR test was conducted to assess short-term recognition memory. Each trial consisted of a familiarization session and a test session. In the familiarization session, each mouse was placed into a testing box (40 × 40 × 40 cm). To reduce anxiety in the animals, the light intensity in the center of the box was set to 100 ± 5 lux. The mouse was allowed to explore freely for 10 min in the box, which contained two identical objects (5 × 5 × 5 cm). One hour later, one familiar object in the box was replaced with a novel object of the same size but different brightness, shape, and texture. In the test session, the mouse was placed back into the box and given 10 min to explore the two objects. Animals that remembered the familiar object spent more time exploring the novel object. Exploration time (snout-to-object distance < 2 cm) was automatically recorded and analyzed using the ANY-maze software (version 7.33, Stoelting Co., USA), based on video tracking. The discrimination index was defined as the ratio of exploration duration around the novel object to the duration around both objects in the test session.

### 2.4 RNA sequencing

Mouse hippocampal tissue was used to extract total RNA employing the Tissue RNA Purification Kit Plus (EZB-RN001-plus). The RNA’s integrity was verified with the RNA Nano 6,000 Assay Kit on an Agilent Bioanalyzer 2,100 system (Agilent Technologies, CA, USA). mRNA was then isolated using poly-T oligo-attached magnetic beads. To enrich for cDNA fragments between 370 and 420 bp, library fragments were purified via the AMPure XP system (Beckman Coulter, Beverly, USA). PCR amplification was carried out using Phusion High-Fidelity DNA Polymerase in combination with Universal PCR primers and an Index (X) Primer; the resulting products were subsequently purified with the AMPure XP system. Library quality was evaluated on the Agilent Bioanalyzer 2,100. Index-coded sample clustering was performed on a cBot Cluster Generation System using the TruSeq PE Cluster Kit v3-cBot-HS (Illumina) following the manufacturer’s protocol. After cluster generation, sequencing on an Illumina Novaseq platform produced 150 bp paired-end reads. Differential gene expression was defined as follows: genes with a *p*-value < 0.05 and fold change >1.5 were considered upregulated, those with a *p*-value < 0.05 and fold change < −1.5 were deemed downregulated, while all other genes were classified as not differentially expressed.

### 2.5 Real-time quantitative polymerase chain reaction (RT-qPCR)

Total RNA was isolated from the dorsal hippocampus using the EZBioscience® Tissue RNA Purification Kit PLUS (EZBioscience, USA) following the manufacturer’s protocol. cDNA synthesis was conducted via reverse transcription with HiScript® II qRT SuperMix (Vazyme, R223, China). RT-qPCR analysis utilized ChamQTM SYBR® Color PCR Master Mix (Vazyme, Q411, China) on a LightCycler® 480 Instrument II (Roche). Gene expression levels were quantified by the 2^−ΔΔCt^ method, with *Gapdh* as the reference (Schmittgen and Livak, 2008). Primer sequences are listed in Supplementary Table S1.

### 2.6 Western blots

Hippocampal tissues were homogenized in chilled RIPA buffer (Beyotime) containing protease/phosphatase inhibitors (Epizyme), followed by 10-min ice incubation and centrifugation (15,000 g, 15 min, 4 °C) to collect soluble fractions. Protein quantification was performed using the BCA method (Thermo Fisher). For immunoblotting, equal protein quantities were resolved via SDS-PAGE and electrotransferred to Polyvinylidene Fluoride (PVDF) membranes. Prior to blocking, membranes were treated with commercial enhancer (Thermo 46,640) to improve signal quality. Nonspecific binding sites were saturated with 5% Bovine Serum Albumin (BSA) / Tris Buffered Saline Tween-20 (TBST) for 1 h at room temperature. Primary antibody incubation was conducted at 4 °C overnight in optimized dilution buffer (Thermo 46,640). After three 10-min TBST washes, membranes were probed with species-matched Horseradish peroxidase (HRP)-conjugated secondary antibodies for 60 min at ambient temperature. Following repeated washes, protein bands were visualized by Enhanced Chemiluminescence (ECL) detection. *β*-actin served as the loading control for quantitative normalization. Complete antibody information is provided in Supplementary Table S2.

### 2.7 Immunofluorescence

Animals were anesthetized using 2.0% isoflurane with 0.30 FiO₂. After achieving unconsciousness, thoracotomy was performed to access cardiac tissue. A cannula was placed into the left ventricular apex for systemic perfusion. Initially, 20 mL chilled Phosphate-Buffered Saline (PBS) was administered, followed by equal volume of 4% Paraformaldehyde (PFA) solution. Following perfusion, cerebral tissues were excised and immersed in fixative at 4 °C for 24 h. Subsequently, specimens were transferred to 30% sucrose-PBS mixture until sedimentation occurred. Tissue blocks were embedded in Optimal Cutting Temperature compound (OCT) medium and sliced coronally at 25 μm thickness using a freezing microtome. For immunohistochemistry, slide-mounted sections underwent three washing cycles with 0.3% PBST. Permeabilization was achieved using 1% Triton X-100 solution for 30 min at room temperature. Background signal suppression was accomplished by 60 min incubation in serum-containing blocking solution. Primary antibody incubation proceeded overnight at 4 °C. After PBS rinses, fluorescent secondary antibodies were applied for 90–120 min under light-protected conditions. Finally, slides were coverslipped using Prolong Glass mounting medium. Antibody specifications are provided in Supplementary Table S2.

### 2.8 Transmission electron microscopy (TEM)

For transmission electron microscopy analysis, fresh tissue samples (~1 mm^3^) were initially fixed in ice-cold 2.5% glutaraldehyde (0.1 M phosphate buffer, pH 7.4) for 2–4 h at 4 °C, followed by three 15-min washes in 0.1 M PBS. Post-fixation was performed using 1% osmium tetroxide (0.1 M PBS) for 2 h at 4 °C under light-protected conditions, with subsequent PBS rinses. Tissue dehydration was achieved through a graded ethanol series (50, 70, 90, 100%) with 15-min intervals, followed by acetone transition. Samples were then infiltrated with epoxy resin using a 1:1 acetone: resin mixture (2 h) and pure resin (overnight) before polymerization at 60 °C for 48 h. Ultrathin sections (70–90 nm) were cut using a diamond knife, collected on copper grids, and sequentially stained with uranyl acetate (15 min) and lead citrate (5 min, CO₂-free environment). All procedures involving hazardous chemicals (particularly osmium tetroxide) were conducted in a fume hood with appropriate personal protective equipment.

### 2.9 Standardized data curation and visualization analysis

All original articles on stress-induced cognitive dysfunction were retrieved from Web of Science Core Collection (WoSCC) in September 2024 (search strategy: Supplementary material). Inclusion criteria: (1) publications from 2000–2024; (2) original research articles (English only). WoSCC was chosen for its comprehensive coverage of >12,000 high-impact journals and reliable citation data. Two investigators independently screened titles/abstracts for relevance. Bibliographic data (authors, institutions, citations, etc.) were extracted and analyzed using Bibliometrix (quantitative metrics) and CiteSpace (co-citation networks, burst detection). All searches were completed on September 2, 2024, to ensure consistency.

### 2.10 Statistical analysis

Statistical evaluations were performed with GraphPad Prism (v10.0, GraphPad Software Inc., USA). Statistical analysis was performed using unpaired t-test and one-way ANOVA with Dunnett’s *post hoc* test. Results are presented as mean ± SEM, and statistical significance was set at *p* < 0.05.

## 3 Results

### 3.1 Aging increases anxiety-like behaviors and impairs short-term recognition memory following restraint stress

**Figure 1:**
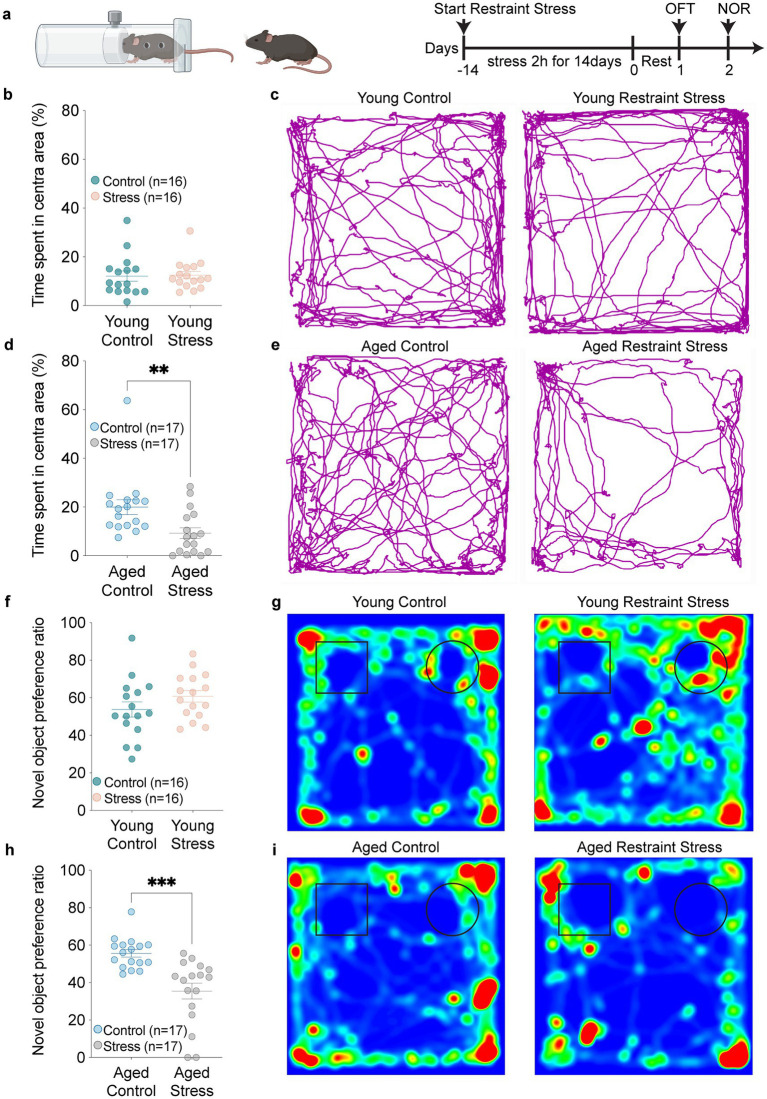
Aging exacerbates stress-induced anxiety and short-term memory recognition deficit. **(a)** Schematic timeline outlining the experimental procedures and behavioral assessments. **(b,c)** Quantitative analysis of time spent in the central zone **(b)** and trackplot **(c)** by young mice in the open field test (OFT). **(d,e)** Quantitative analysis of time spent in the central zone **(d)** and trackplot **(e)** by aged mice in the open field test (OFT). **(f–i)** Quantitative analysis of novel object preference ratio and heatmap by young (**f,g**) and aged (**h,i**) mice in the novel object recognition (NOR). In **b**, **d**, **f**, **h**, data shown are means ± s.e.m. (unpaired t-test), *p < 0.05; **p < 0.01; ***p < 0.001.

To investigate how stressors affect cognitive function, we conducted restrain stress (RS) in 2-and 18-month mice. Then we assessed spontaneous activity and anxiety-like behaviors using the open field test (OFT), and evaluated short-term recognition memory changes with the novel object recognition test (NOR) (Figure 1a). We found that compared to the control groups, young mice displayed no significant differences in locomotor speed (Figures 1b,c), whereas aged RS-exposed mice showed reduced locomotor speed compared to controls (Figures 1d,e). After restraint 2 days, novel object recognition (NOR) testing was performed to test short time memory. In addition, NOR test revealed that aged mice after restraint stress showed significantly reduced novel object exploration time compared to controls, accompanied by a substantially decreased novel object preference ratio. In contrast, young mice subjected to restraint stress exhibited performance comparable to control animals (Figures 1f–i). These findings indicate that aging exacerbates stress-induced memory impairment.

### 3.2 Deeper molecular basis for stress-induced cognitive dysfunction

**Figure 2:**
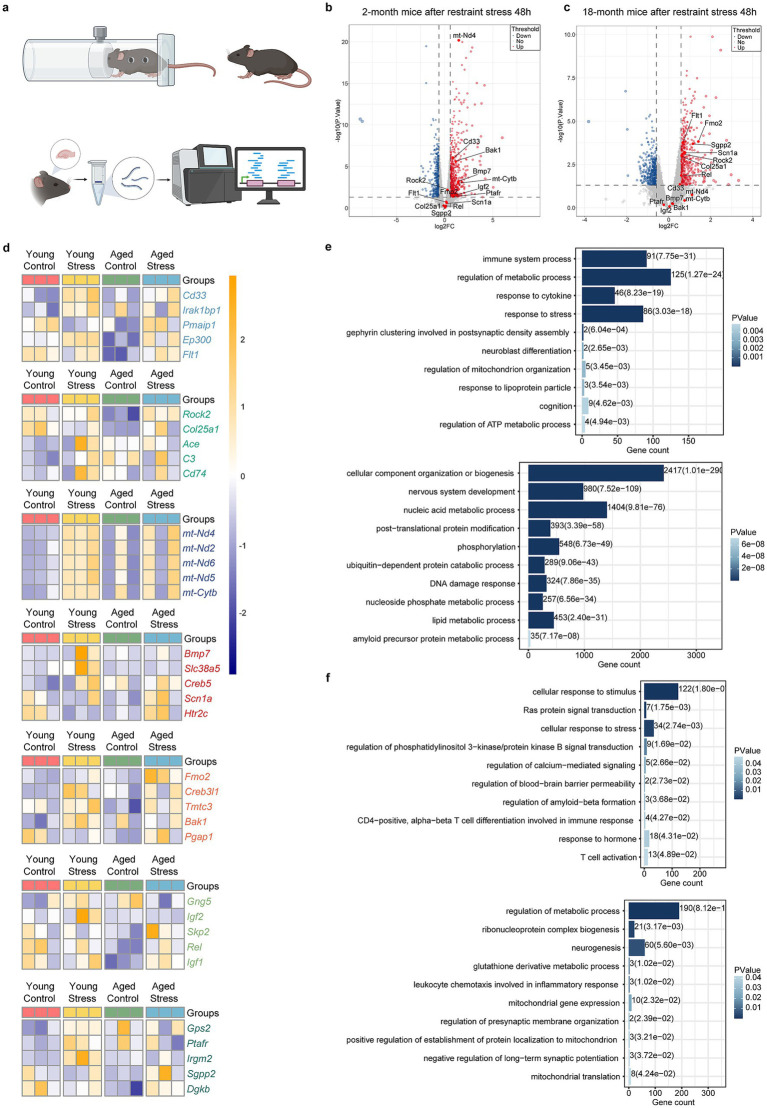
Molecular insight of stress-induced cognitive deficits in aging. **(a)** Flowchart of experimental procedures. **(b,c)** Volcano plots of young mice **(b)** and aged mice **(c)** after restraint stress. Horizontal coordinates represent the fold change in genes expression between the two groups, while vertical coordinates reflect the statistical significance of the difference in metabolite expression. **(d)** Heatmaps showing gene expression in young (left) and aged (right) mice after restraint stress. **(e,f)** Up and down regulated gene-ontology (GO) and KEGG terms in young **(e)** and aged **(f)** mice after restraint stress. The enrichment of genes was analysed by DAVID Bioinformatics Resources, and the Fisher exact test was used for statistical analyses.

To investigate molecular basis for stress-induced cognitive dysfunction, we performed RNA sequencing to identify mRNA changes in the hippocampus of 2- and 18-month mice after restraint stress (Figure 2a). Volcano plots and heatmaps revealed differential changes in multiple functional gene groups following restraint stress during aging, including immune system, RAS signaling, amyloid protein metabolic, ER stress, mitochondrial function, as well as synapse- and neurogenesis process (Figures 2b–d). Functional enrichment analysis of the differentially expressed genes was carried out through the Gene Ontology (GO) and Kyoto Encyclopedia of Genes and Genomes (KEGG) databases. The results revealed that the response to immune system or stress process terms were both upregulated in 2- and 18-month mice. Synapse, Neurogenesis and mitochondrial function terms were up-regulated in 2-month mice and down-regulated in 18-month mice. Amyloid protein metabolic process terms and Lipid metabolic process terms were down-regulated in 2-month mice but up-regulated in 18-month-mice (Figures 2e,f).

**Figure 3:**
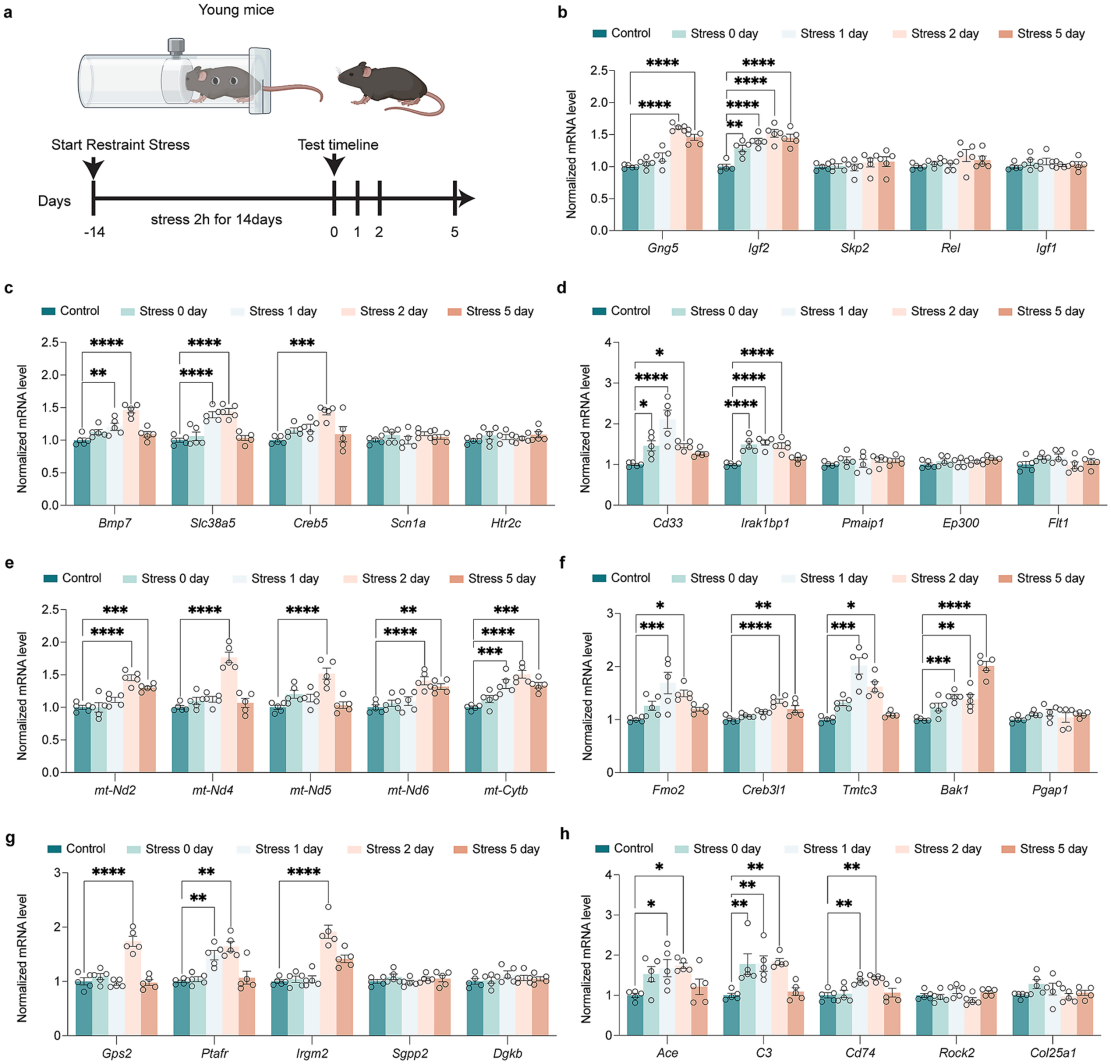
Impact of restraint stress on gene expression in young mice across multiple biological pathways. **(a)** Schematic of the restrain stress in young mice. **(b)** RT-qPCR investigation of transcriptional alterations in genes encoding HPA signal transduction (*Gng5, Igf2, Skp2, Rel, Igf1*) in young mice after restraint stress. **(c)** RT-qPCR investigation of transcriptional alterations in genes encoding synapse and neurogenesis (*Bmp7, Slc38a5, Creb5, Scn1a, Htr2c*) in young mice after restraint stress. **(d)** RT-qPCR investigation of transcriptional alterations in genes encoding immune signal process (*Cd33, Irak1bp1, Pmaip1, Ep300, Flt1*) in young mice after restraint stress. **(e)** RT-qPCR investigation of transcriptional alterations in genes encoding activation of mitochondria function (*mt-Nd2, mt-Nd4, mt-Nd5, mt-Nd6, mt-Cytb*) in young mice after restraint stress. **(f)** RT-qPCR investigation of transcriptional alterations in genes encoding ER stress (*Fmo2, Creb3l1, Tmtc3, Bak1, Pgap1*) in young mice after restraint stress. **(g)** RT-qPCR investigation of transcriptional alterations in genes encoding lipid metabolism process (*Gps2, Ptafr, Irgm2, Sgpp2, Dgkb*) in young mice after restraint stress. **(h)** RT-qPCR investigation of transcriptional alterations in genes encoding amyloid metabolism process (*Ace, C3, Cd74, Rock2, Col25a1*) in young mice after restraint stress. In **b-h**, data are presented as mean ± SEM (n = 5). Statistical significance was determined by one-way ANOVA followed by Dunnett’s *post hoc* test (*p < 0.05, **p < 0.01).

RT-qPCR analysis of these process-related genes confirmed the sequencing results, demonstrating both age-associated upregulation and downregulation patterns following restraint stress (Figure 3a). There were age differences in some gene changes. Some genes were upregulated only after Restraint stress in young mice. In HPA signaling process, *Gng5* and *Igf2* were up-regulated in young mice after restraint stress (Figure 3b). In synapse and neurogenesis process, *Bmp7*, *Slc38a5*, and *Creb5* were up-regulated in young mice after restraint stress (Figure 3c). For immune response processes, *Cd33* and *Irak1bp1* were up-regulated in young mice after restraint stress (Figure 3d). A series of mt-DNA genes (*mt-Nd2, mt-Nd4, mt-Nd5, mt-Nd6, mt-Cytb*) were specifically up-regulated only in young mice, with no changes observed in aged mice after restraint stress (Figure 3e). In ER stress processes, *Fmo2*, *Creb3l1*, *Tmtc3*, and *Bak1* were up-regulated in young mice after restraint stress (Figure 3f). For lipid metabolic processes, *Gps2*, *Ptafr*, and *Irgm2* were up-regulated in young mice (Figure 3g). In amyloid metabolic processes, *Ace, C3*, and *Cd74* were up-regulated in young mice after restraint stress (Figure 3h).

**Figure 4:**
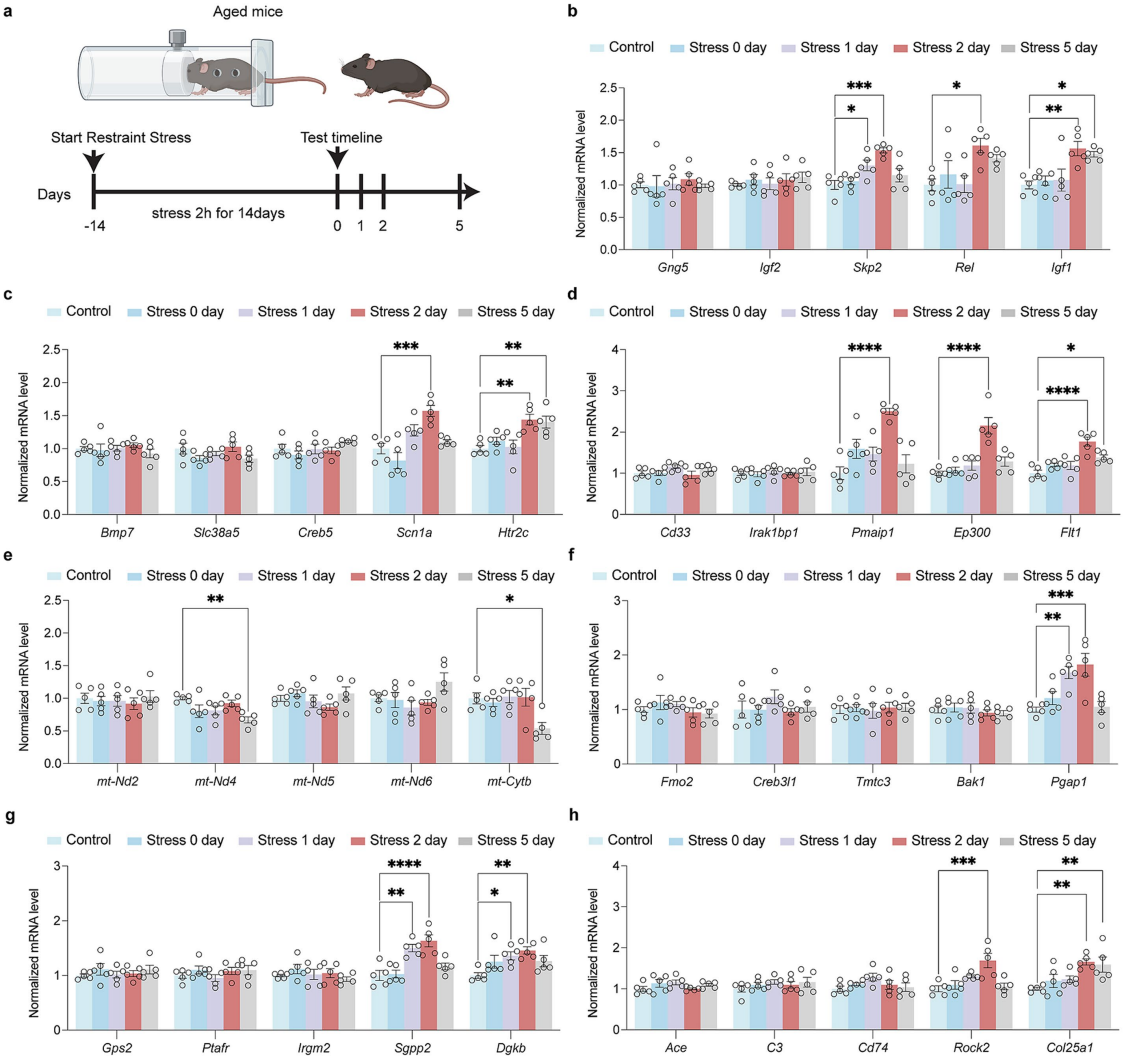
Impact of restraint stress on gene expression in aged mice across multiple biological pathways. **(a)** Schematic of the restrain stress in aged mice. **(b)** RT-qPCR investigation of transcriptional alterations in genes encoding HPA signal transduction (*Gng5, Igf2, Skp2, Rel, Igf1*) in aged mice after restraint stress. **(c)** RT-qPCR investigation of transcriptional alterations in genes encoding synapse and neurogenesis (*Bmp7, Slc38a5, Creb5, Scn1a, Htr2c*) in aged mice after restraint stress. **(d)** RT-qPCR investigation of transcriptional alterations in genes encoding immune signal process (*Cd33, Irak1bp1, Pmaip1, Ep300, Flt1*) in aged mice after restraint stress. **(e)** RT-qPCR investigation of transcriptional alterations in genes encoding activation of mitochondria function (*mt-Nd2, mt-Nd4, mt-Nd5, mt-Nd6, mt-Cytb*) in aged mice after restraint stress. **(f)** RT-qPCR investigation of transcriptional alterations in genes encoding ER stress (*Fmo2, Creb3l1, Tmtc3, Bak1, Pgap1*) in aged mice after restraint stress. **(g)** RT-qPCR investigation of transcriptional alterations in genes encoding lipid metabolism process (*Gps2, Ptafr, Irgm2, Sgpp2, Dgkb*) in aged mice after restraint stress. **(h)** RT-qPCR investigation of transcriptional alterations in genes encoding amyloid metabolism process (*Ace, C3, Cd74, Rock2, Col25a1*) in aged mice after restraint stress. In **b–h**, data are presented as mean ± SEM (n = 5). Statistical significance was determined by one-way ANOVA followed by Dunnett’s post hoc test (*p < 0.05, **p < 0.01).

While there are some genes in the above pathways that are upregulated after Restraint stress in aged mice (Figure 4a). In HPA signaling process, *Skp2*, *Rel*, and *Igf1* were up-regulated in aged mice after restraint stress (Figure 4b). In synapse and neurogenesis process, *Scn1a* and *Htr2c* were up-regulated in aged mice (Figure 4c). For immune response processes, *Pmaip1*, *Ep300*, and *Flt1* were up-regulated in aged mice (Figure 4d). A series of mt-DNA genes (*mt-Nd2, mt-Nd4, mt-Nd5, mt-Nd6, mt-Cytb*) were not up-regulated in aged mice after restraint stress (Figure 4e). In ER stress processes, *Pgap1* was up-regulated in aged mice (Figure 4f). For lipid metabolic processes, *Sgpp2* and *Dgkb* were up-regulated in aged mice following restraint stress (Figure 4g). In amyloid metabolic processes, *Rock2* and *Col25a1* were up-regulated in aged mice (Figure 4h).

### 3.3 Aging exacerbates stressor-induced synaptic and mitochondrial impairments

**Figure 5:**
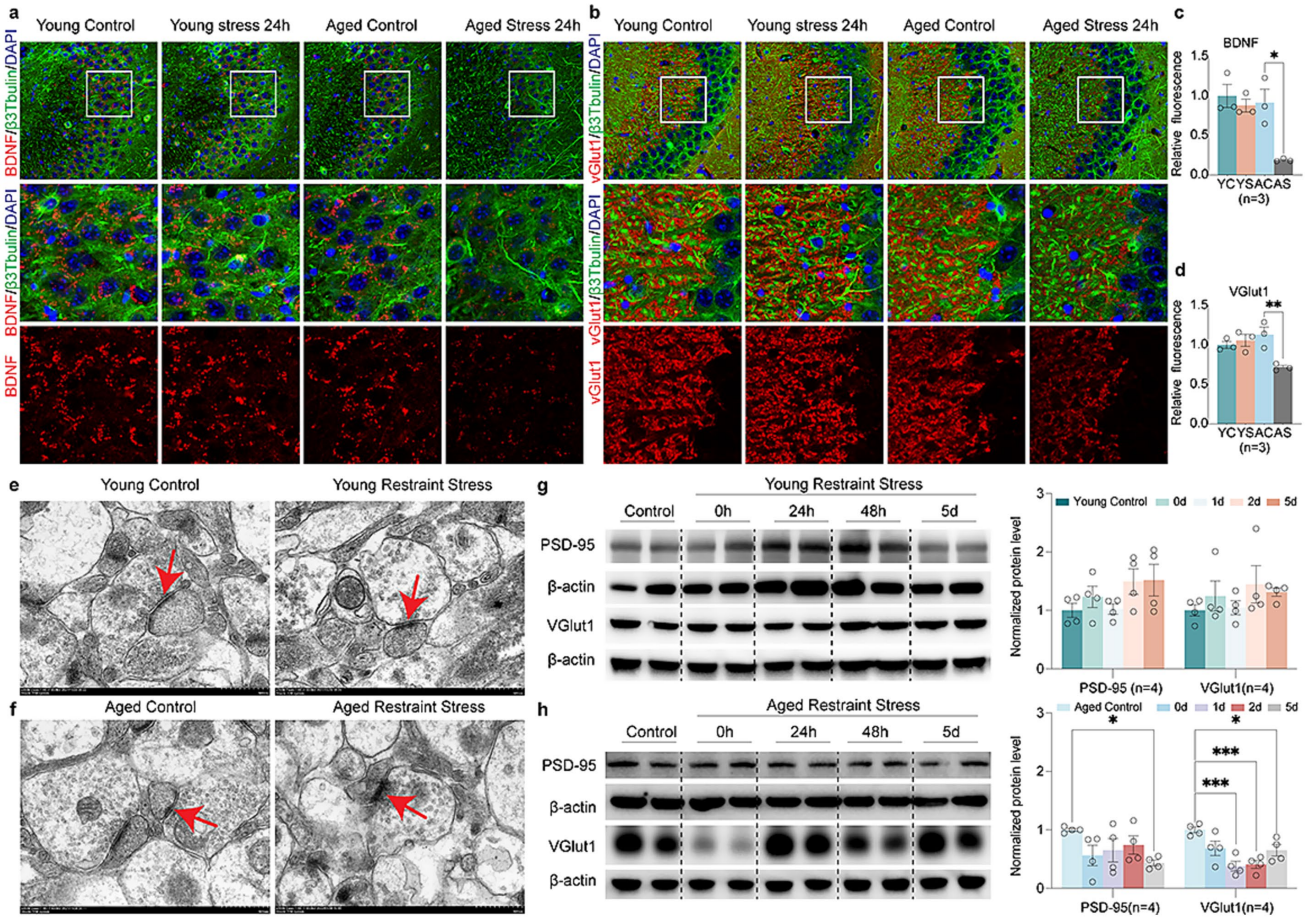
Aging accelerates stress-mediated synaptic protein degradation. **(a,b)** Fluorescent images of young and aged mice expressing the synaptic protein BDNF **(a)** and VGlut1 **(b)** after restraint stress 48 h. **(c,d)** Quantitative analysis of young and aged mice expressing the synaptic protein BDNF **(c)** and VGlut1 **(d)** after restraint stress 48 h. **(e,f)** Age-related TEM alterations in hippocampal synapses of young **(e)** vs. aged **(f)** mice post 48 h restraint stress. **(g,h)** Representative western blots and quantitative analysis of synaptic protein (BDNF, VGlut1) levels in young **(g)** and aged **(h)** mice. *β*-actin expression is used as a reference. In **c**, **d**, **f**, **g**, data shown are means ± s.e.m. (one-way ANOVA with Dunnett’s test), *p < 0.05; **p < 0.01; ***p < 0.001. Scale bars, 10 μm for **a**, **b**, 500 nm for **d**, **e**.

**Figure 6:**
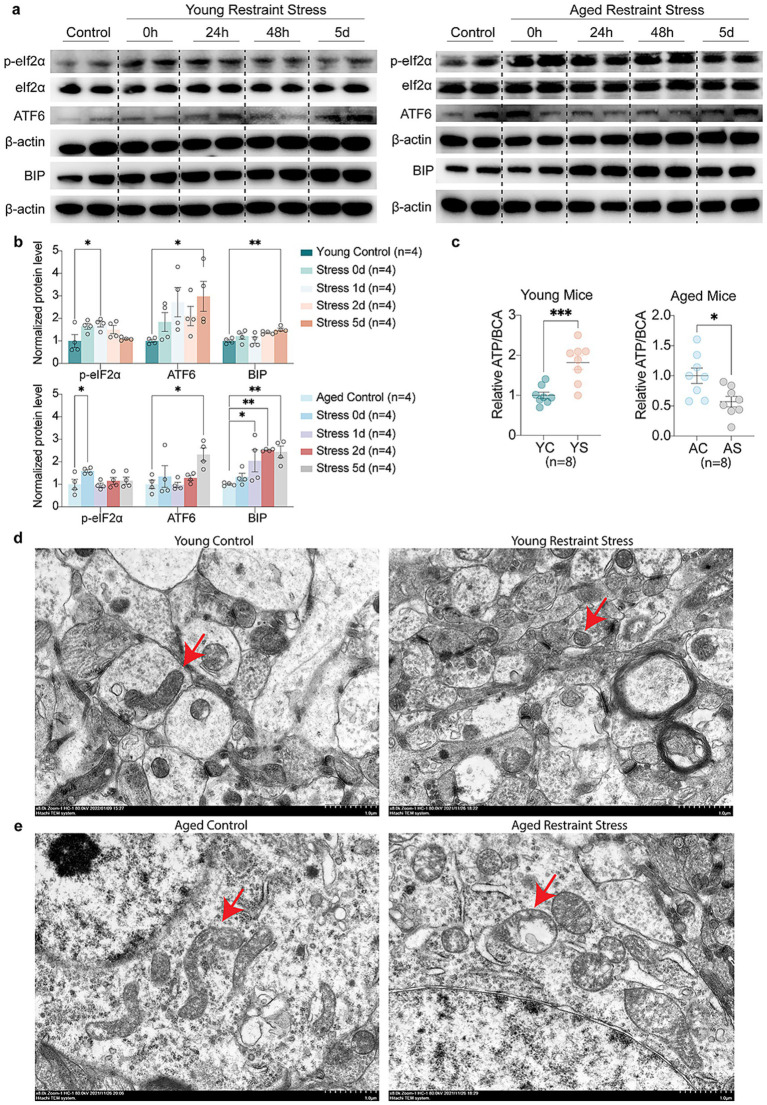
Aging accelerates stress-mediated Endoplasmic Reticulum stress and mitochondrial dysfunction. **(a,b)** Representative western blots **(a)** and quantitative analysis **(b)** of Endoplasmic Reticulum stress markers (p-eIF2α, ATF6, BIP) levels in young and aged mice. β-actin expression is used as a reference. **(c)** Quantitative analysis of ATP levels in young and aged mice hippocampal. BCA levels expression is used as a reference. **(d,e)** Age-related TEM alterations in hippocampal mitochondria of young **(d)** vs. aged **(e)** mice post 48 h restraint stress. In **b**, **c**, data shown are means ± s.e.m. (one-way ANOVA with Dunnett’s test, Unpaired *t*-test), *p < 0.05; **p < 0.01; ***p < 0.001. Scale bars, 1.0 μm for **d**, **e**.

To examine how stressors affect synaptic function during aging, immunofluorescence analysis revealed Brain-Derived Neurotrophic Factor (BDNF) and Vesicular Glutamate transporter 1 (VGlut1) up-regulated in young mice, which was absent in aged mice post-restraint stress (Figures 5a–d). The transmission electron microscopy (TEM) results revealed that the synaptic structures, including the synaptic cleft and synaptic vesicles, remained intact in the hippocampal neurons of young mice following restraint stress (Figure 5e). In contrast, 48 h after restraint stress, aged mice exhibited significant disruption of synaptic ultrastructure, characterized by a narrowed synaptic cleft and a reduced number of synaptic vesicles (Figure 5f). Quantitative Western blot analysis demonstrated that, unlike young mice, aged mice exhibited a time-dependent decrease in the synaptic proteins Postsynaptic Density Protein-95 (PSD-95) and VGlut1 following restraint stress (Figures 5g,h). These findings suggest that aged mice are more susceptible to synaptic dysfunction following restraint stress compared to adult mice. We further investigated the impact of stressor on ER stress and mitochondrial function during aging. Western blot analysis revealed that both young and aged mice developed ER stress following restraint stress, but the response was more pronounced in aged mice. Specifically, aged mice exhibited elevated phosphorylation of eIF2α, along with increased levels of Activating Transcription Factor 6 (ATF6) and Immunoglobulin Heavy Chain-Binding Protein (BIP) (Figures 6a,b). Additionally, Adenosine Triphosphate (ATP) assays demonstrated a significant reduction in hippocampal ATP content in aged mice compared to young mice after restraint stress (Figure 6c). TEM analysis revealed that hippocampal mitochondria in young mice became rounded but maintained intact double-membrane structures 48 h after restraint stress (Figure 6d). In contrast, aged mice exhibited pronounced mitochondrial swelling, accompanied by structural damage including disrupted double membranes and loss of cristae following the same stress duration (Figure 6e).

### 3.4 Validation of research fronts and hot spots in stress-induced cognitive dysfunction

**Figure 7:**
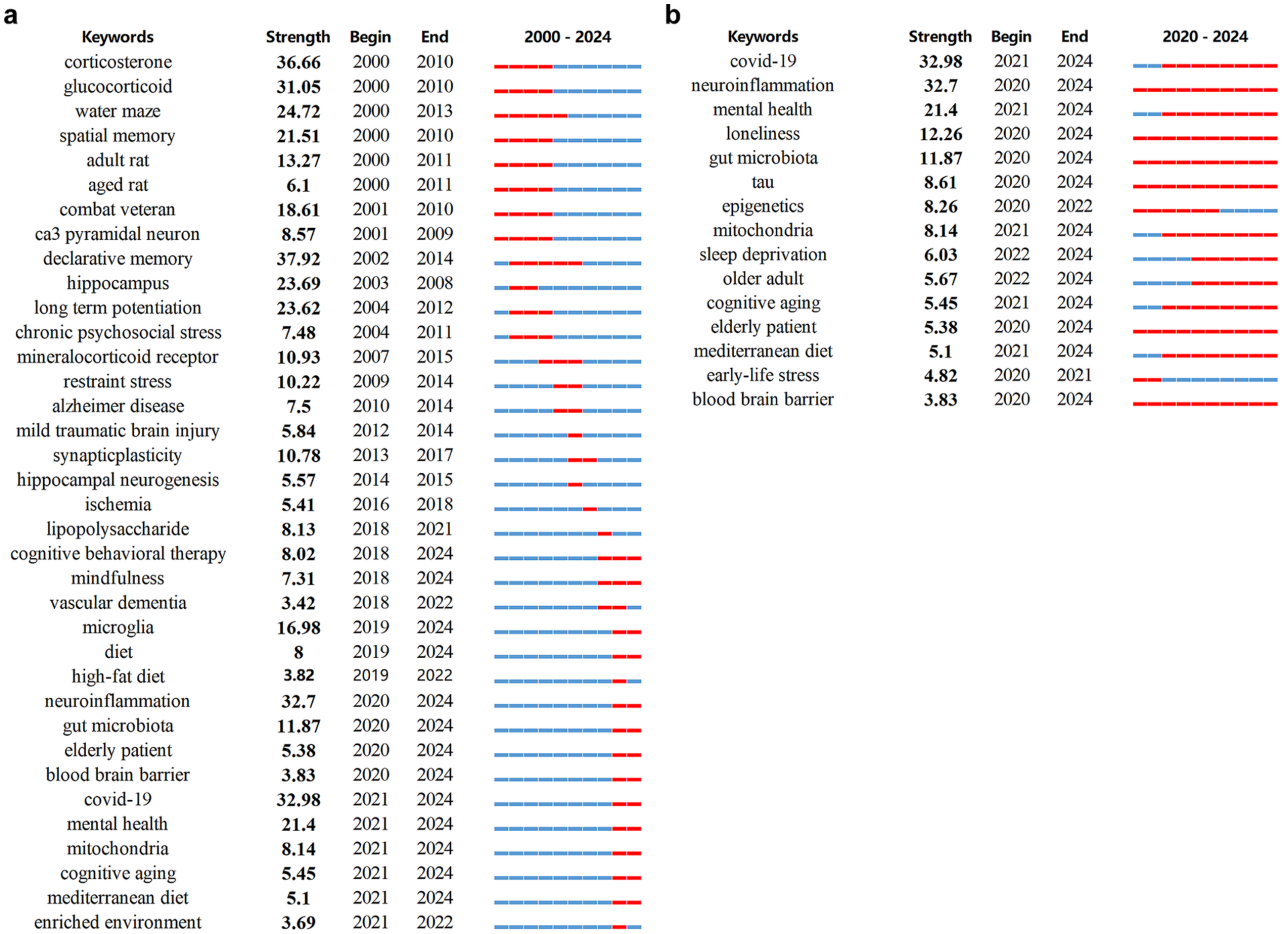
Burst keywords detected between 2002-2024 **(a)** and 2020-2024 **(b)**. The burst period is highlighted in red on the blue timeline, marking its start, end, and duration.

Finally, we utilized burst detection analysis to capture rapid surges of interest in specific research topics related to stress-induced cognitive dysfunction. This technique, implemented via CiteSpace, was applied to both keyword and reference data to identify temporal patterns of emerging scientific focus. Keyword burst detection effectively highlighted emergent hotspots in the field. After filtering out terms with minimal academic relevance, we identified some keywords with the strongest citation bursts, reflecting evolving research trends on stress-related cognitive decline (Figure 7a). Notably, corticosterone exhibited the highest burst strength (36.66), peaking during the 2002–2014 period, indicative of early emphasis on memory systems vulnerable to stress. Early-stage research predominantly focused on the hypothalamic–pituitary–adrenal (HPA) axis, with keywords such as corticosterone and glucocorticoid, as well as on hippocampal-dependent processes including spatial memory and neurogenesis, which are critical to synaptic plasticity and cognitive resilience in aging. In contrast, recent bursts reveal a shift toward mechanistic and translational investigations, highlighting terms such as microglia, neuroinflammation, gut microbiota, tau, epigenetics, mitochondria, ketogenic diet, COVID-19 survivors, and subjective cognitive decline. These trends reflect a broader systems-level approach to understanding how neuroimmune and metabolic factors contribute to cognitive vulnerability in older adults (Figure 7b). Importantly, several burst keywords—adult rat, aged rat, older adult, elderly patient, and cognitive aging—persist across the entire time span, emphasizing the central and enduring role of aging in mediating susceptibility to stress-induced cognitive impairment. The results of burst keyword detection showed direct correspondence with transcriptomic findings (Table 1). For instance, the keyword corticosterone (2000–2010) mapped onto enriched pathways such as immune system processes, stress responses, and Ras protein signaling, together with age-dependent changes in genes including *Gng5*, *Igf2*, and *Skp2*. Likewise, the keyword mitochondria (2021–2024) corresponded to enrichment of mitochondrial pathways, including regulation of ATP metabolism and mitochondrial gene expression, along with alterations in mtDNA. These findings were further corroborated by ATP assays and transmission electron microscopy, which confirmed age-dependent declines in mitochondrial morphology and function following stress. These findings support the notion that age-related biological changes, including altered neuroinflammatory responses and mitochondrial dysfunction, are key contributors to the pathogenesis of cognitive decline following stress exposure.

**Table 1:** Cross-mapping of bibliometric and experimental data.

Keywords (bibliometric)	Key pathways (experimental data)	Key genes (experimental data)
Corticosterone (2000–2010) Glucocorticoid (2000–2010)	Immune system process, Ras protein signal transduction	*Gng5, Igf2, Skp2, Rel, Igf1*
Synapticplasticity (2013–2017) Hippocampal neurogenesis (2014–2015)	Regulation of presynaptic membrane organization, Neurogenesis, Negative regulation of long-term synaptic potentiation	*Bmp7, Slc38a5, Creb5, Scn1a, Htr2c*
Early-life stress (2020–2021)	Response to stress, Ribonucleoprotein complex biogenesis	*Fmo2, Creb3l1, Tmtc3, Bak1, Pgap1*
Mediterranean diet (2021–2024)	Lipid metabolic process	*Gps2, Ptafr, Irgm2, Sgpp2, Dgkb*
Mitochondria (2021–2024)	Regulation of mitochondrion organization, Regulation of ATP metabolic process, Mitochondrial gene expression, Mitochondrial translation	*mt-Nd2, mt-Nd4, mt-Nd5, mt-Nd6, mt-Cytb*
Tau (2020–2024)	Amyloid precursor protein metabolic process, Regulation of amyloid-beta formation	*Ace, C3, Cd74, Rock2, Col25a1*

## 4 Discussion

### 4.1 Overview

Stress is an unavoidable part of life, and its impact on brain function, particularly in the hippocampus, is well documented. However, different types of stressors exert distinct effects on cognition. This study integrates transcriptome sequencing and bibliometric analysis to complement each other from two dimensions—molecular mechanisms and macro-level academic trends. Such integration not only enables micro-level discoveries to be rapidly contextualized within the broader research landscape, but also allows bibliometric hotspots to guide deeper interrogation of transcriptomic data, thereby identifying potential novel targets. In this way, the combined approach significantly enhances both the depth and breadth of understanding stress-induced cognitive dysfunction during aging.

Transcriptomic profiling revealed age-dependent molecular alterations in stressed mice, including dysregulation of synaptic plasticity, impaired neurogenesis, HPA axis hyperactivation, neuroimmune activation, ER stress, mitochondrial dysfunction, and amyloid metabolic abnormalities. Bibliometric analysis complemented these findings by highlighting emerging research priorities, such as healthy aging models, amyloid-related mechanisms, dietary interventions (e.g., the Mediterranean diet), and organelle stress responses. This integrative approach enhances our understanding of both biological mechanisms and the evolving direction of research in this field.

### 4.2 Trends in stress induced-cognitive dysfunction research

Historical work by McEwen established the foundation for understanding stress’s impact on the hippocampus and the role of glucocorticoids (McEwen et al., 1968). This foundational work spurred subsequent research into how Excessive HPA axis activation leads to abnormally high cortisol levels. Consistent with these findings, while both young and aged mice exhibited RAS system activation following restraint stress, the specific genes upregulated during aging showed distinct patterns. Our RT-qPCR analysis demonstrated a differential stress response across age groups: young mice showed significant upregulation of *Gng5, Skp2,* and *Igf2*, whereas aged mice predominantly upregulated *Igf1* and *Rel*. Notably, *Igf2* expression displays an age-dependent decline in the brain (Xia et al., 2019; Argyraki et al., 2019; Adamis et al., 2020), which may contribute to reduced synaptic plasticity in aging. Conversely, *Rel* not only synergizes with RAS signaling but also promotes amyloidogenic processing through BACE1 upregulation (Jaafaru et al., 2019), potentially exacerbating Alzheimer’s-like pathology in stressed aged mice. Collectively, these findings highlight the intricate interplay between inflammation, stress responses, and RAS signaling during aging, offering novel insights for developing anti-aging and neuroprotective strategies. In 2000, McEwen provided further evidence that chronic stress inhibits neurogenesis in the hippocampus, leading to neuronal loss and synaptic damage (Carroll and Malenka, 2000). This work laid the groundwork for understanding the link between stress, neurogenesis, and cognitive impairment. Consistent with previous studies, our findings demonstrate that aging exacerbates synaptic impairment in mice following restraint stress, as evidenced by reduced expression of synaptic proteins (PSD-95, VGlut1 and BDNF) and ultrastructural synaptic damage. It expanded understanding emphasizes the interplay between neurogenesis and synaptic health in maintaining cognitive function under stress. Hypothesized nearly a century ago and continues to be a major research focus (Mondelli et al., 2017). These immune-mediated processes ultimately disrupt memory formation and learning (Gaikwad et al., 2024; Zhang et al., 2023; Piirainen et al., 2017; Annane and Sharshar, 2015). Our transcriptomic data revealed similar change. Consistent with our transcriptomic sequencing results, bibliometric analysis also revealed parallel patterns. Burst keyword detection showed that between 2000 and 2010, corticosterone and glucocorticoid ranked among the top terms, with strengths of 36.66 and 31.05, respectively. Similarly, our transcriptomic data demonstrated enrichment of the Ras protein signal transduction pathway, with age-dependent differences in Ras-related gene expression: restraint stress predominantly upregulated *Igf2* in young mice, whereas *Igf1* was increased in aged mice. In line with these findings, the bibliometric appearance of adult rat and aged rat (2000–2011) suggested that aging plays a critical role in stress-induced cognitive impairment. Between 2013 and 2017, synaptic plasticity and neurogenesis emerged as mainstream research focuses and have continued to develop, culminating around 2020 in a surge of burst keywords related to neuroinflammation and microglia. Burst keywords in synaptic plasticity and neurogenesis are consistent with our findings of reduced PSD-95, VGlut1, and BDNF expression and impaired hippocampal ultrastructure. Together, these findings demonstrate that stress—whether through peripheral immune stimulation or direct central nervous system activation—induces microglial reactivity, pro-inflammatory cytokine release, and subsequent synaptic dysfunction.

### 4.3 Research fronts of the stress induced-cognitive dysfunction

Recent research has increasingly underscored the critical involvement of cellular and organelle stress pathways in cognitive dysfunction. Stress-induced perturbations in energy metabolism and protein homeostasis primarily mediated through mitochondrial dysfunction and endoplasmic reticulum (ER) stress significantly increase neuronal vulnerability. Compelling evidence indicates that mitigation of ER stress, particularly through inhibition of the CHOP signaling pathway, can improve cognitive outcomes (Civas et al., 2017; Venkataraman et al., 2022). Our investigations reveal that aging differentially modulates restraint stress-induced ER stress responses: while suppressing the upregulation of protective ER stress-related genes (*Fmo2, Creb3l1, Tmtc3, Bak1*), it promotes the induction of *Pgap1* change associated with cognitive decline (Yuan et al., 2023). Notably, existing studies have confirmed that *Fmo2* can reduce the accumulation of misfolded proteins during ER stress, whereas *Creb3l1* facilitates proper protein folding by endoplasmic reticulum stress (Jaafaru et al., 2019; Yuxiong et al., 2023; Liu et al., 2023). *Bak1* contributes to the clearance of ER stressors by promoting autophagy and maintaining the function of mitochondria-associated membranes (Guo et al., 2022; Xue et al., 2023). Upregulation of *Pgap1*, on the other hand, may exacerbate ER stress by disrupting GPI-anchored protein processing, impairing synaptic protein homeostasis, and depleting ER repair capacity, ultimately promoting neuronal injury and cognitive decline (Hong et al., 2024). Consistent with these studies, Western blot analysis indicated that both young and aged mice exhibited upregulation of ER stress markers ATF6 and BIP, along with increased phosphorylation of eIF2α following restraint stress. However, the differing degrees of ER stress suggest that distinct downstream responses may be triggered in each group. Mitochondrial dysfunction has been firmly established as a driver of neurodegenerative disease progression (Dvela-Levitt et al., 2019). Our GO enrichment analysis demonstrates that restraint stress in young mice primarily upregulates DEGs involved in mitochondrion organization and ATP metabolic regulation, whereas aged mice show downregulation of mitochondrial gene expression and translation pathways. This age-dependent divergence was further corroborated by RT-qPCR: while restraint stress increased mtDNA expression in adult mice, this adaptive response was absent in aged counterparts, underscoring the pivotal role of mitochondrial homeostasis in cognitive preservation. Consistent with the transcriptomic sequencing results, we also observed a decline in hippocampal ATP levels in aged mice following restraint stress, whereas no such decrease was detected in young mice. Neuronal recovery after restraint stress requires substantial energy, and the reduced ATP supply may be one of the key factors contributing to the increased vulnerability of aged mice to cognitive impairment (Owen and Sunram-Lea, 2011; van de Wal et al., 2022). Transmission electron microscopy (TEM) further revealed that restraint stress in aged mice was accompanied by widespread mitochondrial morphological damage. Phosphorylated tau deposition is known to exacerbate both mitochondrial dysfunction and ER stress. As highlighted by Zhongcong Xie’s work, targeting tau hyperphosphorylation represents a promising therapeutic strategy to mitigate anesthesia-induced neural network impairments and retrograde amnesia in Alzheimer’s disease (Chen et al., 2024). In this study, RT-qPCR analysis further reveal that aging alters stress responses by suppressing *Ace*, *C3*, and *Cd74* induction while promoting *Rock2* and *Col25a1* up-regulated changes of particular significance given that elevated *Ace* expression reduces tau amyloid deposition whereas *Col25a1* accumulation accelerates it (Kunkle et al., 2019; Bellenguez et al., 2022; Swanson et al., 2020; Zhong et al., 2023; Litvinchuk et al., 2018; Drummond et al., 2022; Tanaka et al., 2014).

In bibliometric terms, combining these findings with burst detection allowed us to identify and clarify research fronts and emerging concepts at both structural and temporal levels. Burst keyword detection over the past 5 years highlighted mitochondria, tau, Mediterranean diet, and cognitive aging. Notably, these themes correspond closely with our experimental observations: the burst in mitochondria parallels our evidence of mitochondrial gene downregulation, reduced ATP levels, and TEM-detected structural damage in aged mice following restraint stress; while tau and lipid metabolism align with our findings of *Col25a1* upregulation and *Ace* downregulation, changes that are closely linked to amyloid and tau pathology. The research front of a field refers to a set of citing articles in bibliometric terms. Through the analysis of word profiles derived from articles citing a particular reference cluster, co-citation labels are generated, facilitating the identification and interpretation of key themes within the research front. In this study, we provide a concise discussion on how these emerging research fronts have advanced the field in promising new directions and highlight potential avenues for future exploration. In our analysis, cluster labels were assigned using word profiles drawn from citing articles with a co-cited reference cluster. The 12 major cluster labels included subjective cognitive decline, amyloid pathology, stress experience, hydroxysteroid dehydrogenase type, memory retrieval, chronic psychosocial stress, coping strategy, posttraumatic stress disorder, post-intensive care syndrome, sedation protocol, prenatal cocaine exposure and cognitive health (Supplementary Figure S1a). Supplementary Figure S1b presents a timeline overview of each co-citation cluster. Cluster #0 (subjective cognitive decline), Cluster #1 (Amyloid pathology), Cluster #3 (hydroxysteroid dehydrogenase type), Cluster #9 (post-intensive care syndrome) and Cluster #12 (cognitive health) showed the latest citation bursts, reflecting the emerging research trends within the field of stress-induced cognitive dysfunction. In our analysis, VOSviewer categorized the keywords of the stress-induced cognitive dysfunction field into four major clusters, representing neurobiological mechanisms, chronic mental health issues, neurodegenerative diseases, and trauma-related psychological disorders (Supplementary Figure S1c). The red cluster mainly related to neurobiological mechanisms underlying memory and learning, which suggested a focus on how stress impacts neural circuits and processes involved in memory formation, particularly in areas of the brain associated with cognitive and emotional regulation. Corticosterone and amygdala further emphasized the role of stress hormones and fear emotion in memory processing. The green cluster centers on chronic mental health issues, particularly depression, indicating a focus on the effects of low-intensity, chronic, ongoing trauma stress on mental health in aging populations. This includes research on how depression and related symptoms contribute to cognitive impairment and quality of life in elderly. The yellow cluster is focused on chronic neurodegenerative disease Alzheimer’s disease (AD). The emergence of microglia, astrocyte and endoplasmic reticulum stress suggested that the concept of stress has been refined from the level of individual to the level of cell. Research here focused on how stress can accelerate neurodegenerative processes through inflammation. The blue cluster covers transient trauma stress but lasting damage-related psychological disorders, PTSD. This cluster includes studies examining how high-intensity, instantaneous trauma stress-related disorders impair specific cognitive domains. The understanding of stress-induced cognitive dysfunction in AD, PTSD, and depression has expanded with evidence pointing to the roles of HPA axis dysregulation, neuroinflammation, synaptic plasticity changes, neurotrophic factors, and neurotransmitter imbalances, disruption of protein homeostasis and endoplasmic reticulum stress. Future research should focus on therapeutic interventions targeting key mechanisms such as inflammation, neurogenesis, and HPA axis modulation to mitigate cognitive decline (Supplementary Figure S1d).

### 4.4 Current limitations and future challenges

While our study integrates transcriptomic sequencing, molecular validation, and bibliometric analysis, several limitations should be acknowledged. The experimental findings are based on a restraint stress model in mice, which, despite its utility, may not fully capture the complexity of human stress responses and age-related cognitive impairment. In addition, our bibliometric analysis was restricted to English-language publications indexed in the Web of Science Core Collection, potentially omitting relevant studies from other databases or languages. Future research incorporating multi-omics approaches, cross-species validation, and clinical samples will be essential to enhance the robustness and translational relevance of these findings.

## 5 Conclusion

This study integrates transcriptomics, molecular biology, and bibliometrics to profile the research landscape of stress-induced cognitive dysfunction in aging. Understanding has shifted from HPA axis dysregulation and hippocampal damage to molecular mechanisms. While early work emphasized impaired neurogenesis, recent studies show chronic stress also disrupts neurotransmitter balance, synaptic plasticity, and mitochondrial function. Future research may incorporate proteomics and epigenomics to deepen insights into age-related cognitive decline.

## Data Availability

The RNA-seq data generated in this study have been deposited in the NCBI Sequence Read Archive (SRA) under BioProject accession PRJNA1345168. The associated BioSample accessions are SAMN5263241–SAMN5263252, and the SRA accession numbers are SRR3578196–SRR3578207. The data will be publicly available on October 15, 2026. Until release, the data can be accessed upon reasonable request to the corresponding author. The processed mRNA-seq data is included as table1.xlsx in Supplementary materials.
