# Supplementary material for: Stressors-induced cognitive dysfunction during aging: mechanisms and future challenges
Source: Front Aging Neurosci. 2025 Oct 22;17:1630982. doi: 10.3389/fnagi.2025.1630982 (PMC12585983; doi:10.3389/fnagi.2025.1630982)
Supplement: Supplementary file 1 [file Data_Sheet_1.pdf]

Supplementary Material

1 Supplementary Data

Data Collection and Analysis

All relevant original articles on stress-induced cognitive dysfunction were retrieved from the Web of Science Core Collection (WoSCC), with detailed search strategies provided in the Supplementary Material. The literature search was conducted in September 2024, applying the following inclusion criteria: (i) publication period from January 2000 to September 2024, (ii) document type restricted to original articles due to their higher academic novelty and impact, and (iii) English-language publications. WoSCC was selected as the primary data source given its reputation as a leading academic database, indexing over 12,000 high-impact journals and providing comprehensive citation records essential for bibliometric analysis. Bibliographic data—including titles, authors, affiliations, publication years, journals, citation counts, and full references—were extracted and stored. Two researchers independently screened titles and abstracts to ensure relevance, and eligible records were saved in .txt format for further analysis. To minimize discrepancies from database updates, all searches and data extraction were completed on September 2, 2024.

For bibliometric assessment, CiteSpace and Bibliometrix (an R package) were employed: Bibliometrix facilitated quantitative analyses of scientific output, authorship, institutional contributions, and geographic distributions, while CiteSpace enabled network visualization, co-citation clustering, and burst detection to identify emerging trends. This integrated approach ensured robust evaluation of the field's intellectual structure and dynamic patterns

2 Supplementary Figures and Tables

2.1 Supplementary Figures

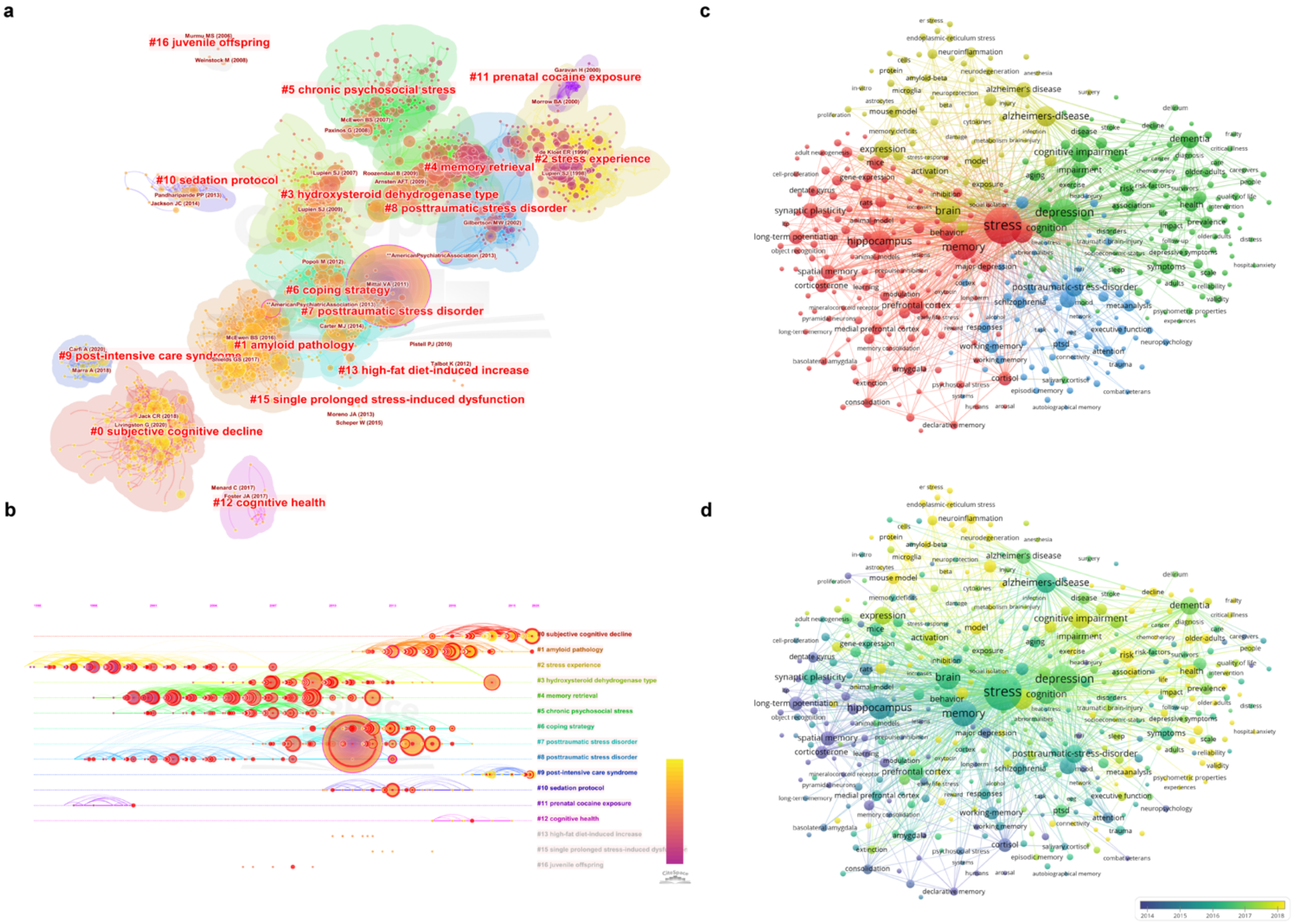

Supplementary Figure 1. Co-citation analysis of articles and keyword co-occurrence network in stress-induced cognitive dysfunction research.

(a, b) Co-citation analysis of articles from the past two decades. Co-citation clustering of articles on stress-induced cognitive dysfunction**(a)**. The timeline view of co-citation clusters with cluster labels shown on the right **(b)**. **(c, d)** Analysis of the co-occurrence network for keywords (n = 376) appeared a minimum of 20 times. Network view with four clusters assigned with different colors **(c)**. Overlay view with color representing the average publication year for each keyword **(d)**.

2.2 Supplementary Tables

Supplementary table 1 Primers

Gene Name	Forward primer sequence (5’ → 3’)	Reverse primer sequence (5’ → 3’)
Gng5	CTCAACCGCGTGAAGGTTTC	GGTCTGAAGGGATTCGTACTTG
Igf2	GTGCTGCATCGCTGCTTAC	ACGTCCCTCTCGGACTTGG
Skp2	ATGGACTGCTCTCAAACCTCG	CCTGGAAAGTTCTCCCGACTAA
Rel	AGAGGGGAATGCGGTTTAGAT	TTCTGGTCCAAATTCTGCTTCAT
Igf1	GTGAGCCAAAGACACACCCA	ACCTCTGATTTTCCGAGTTGC
Bmp7	ACGGACAGGGCTTCTCCTAC	ATGGTGGTATCGAGGGTGGAA
Slc38a5	CTACAGGCAGGAACGCGAAG	GGTTGAACACTGACATTCCGA
Creb5	AGGATCTTCTGCCGTCTTGAT	GCGCAGCCTTCAGTCTCAT
Scn1a	TCAGAGGGAAGCACAGTAGAC	TTCCACGCTGATTTGACAGCA
Htr2c	CTAATTGGCCTATTGGTTTGGCA	CGGGAATTGAAACAAGCGTCC
Cd33	CCGCTGTTCTTGCTGTGTG	AAGTGAGCTTAATGGAGGGGTA
Irak1bp1	AGCCGAGGTCTGCATTACATT	TGGCAGTCTGGATAACTGATGA
Pmaip1	GCAGAGCTACCACCTGAGTTC	CTTTTGCGACTTCCCAGGCA
Ep300	TTCAGCCAAGCGGCCTAAA	CGCCACCATTGGTTAGTCCC
Flt1	TGGCTCTACGACCTTAGACTG	CAGGTTTGACTTGTCTGAGGTT
mt-Nd2	TCACCCTTGCCATCATCT	TTGAGGCTGTTGCTTGTG
mt-Nd4	AACGGATCCACAGCCGTA	AGTCCTCGGGCCATGATT
mt-Nd5	TTCCTAACAGGGTTCTACTC	GGTCTGGGTCATTTTCGT
mt-Nd6	CTACCCCAATCCCTCCTT	GGTTTGGTGGATCGTTTT
mt-Cytb	CCCATTCATTATCGCCGC	GGGTGTTGAGGGGGTTAGC
Fmo2	CAGCAGCCAAACGAACATCC	CTCCTGCAACTTTCCCTCCT
Creb3l1	GCCCTGGGAAACAAGCTGT	AGCTGAGTCATTTCTCCTGGG
Tmtc3	CGCCTGCTACTGGAACAGC	GTTCGCTTTGAACTCGGTCTT
Bak1	CAACCCCGAGATGGACAACTT	CGTAGCGCCGGTTAATATCAT
Pgap1	CTTCTCCTGGTGTAGAGGCG	GATTCCAGAGGTTGACTGAGTG
Gps2	GGAAGAAAAACGGAGGCGAAA	GAGTACCTGGGCGATTGTGTC
Ptafr	CTGAGTTTCGATACACGCTCTT	TTGCTTTTGATTGTTCGAGCAG
Irgm2	GGCAGTTGAGTCACCTGAGG	CCCCTTCTTTCACGGCAGT

Sgpp2	TTCACCCACTGGAATATCGACC	AAGTCTCACAACGGGAGGAAA
Dgkb	CCGCTTCTAGTTTTTGTCAACCC	AGACTGTAAACCTGACGAGGATT
Ace	AGGTTGGGCTACTCCAGGAC	GGTGAGTTGTTGTCTGGCTTC
C3	CCAGCTCCCCATTAGCTCTG	GCACTTGCCTCTTTAGGAAGTC
Cd74	AGTGCGACGAGAACGGTAAC	CGTTGGGGAACACACACCA
Rock2	TTGGTTCGTCATAAGGCATCAC	TGTTGGCAAAGGCCATAATATCT
Col25a1	GTCCCAAAGGTGATACAGGAGA	CCTTGAGGACCAATGAAGCCT
Gapdh	AGGTCGGTGTGAACGGATTTG	TGTAGACCATGTAGTTGAGGTCA

Supplementary table 2 Antibodies Information

Antibody	Information ID	Product
Goat Anti Rabbit IgG Alexa Fluor 488	ab150061	Abcam
Goat Anti Rabbit IgG Alexa Fluor 594	ab150064	Abcam
Goat Anti Mouse IgG Alexa Fluor 488	ab150109	Abcam
Goat Anti Mouse IgG Alexa Fluor 594	ab150112	Abcam
HRP Anti-beta Actin	Ab49900	Abcam
eIF2α (D7D3) Rabbit mAb	5324T	Cell Signaling Technology
phospho-eIF2α (Ser51) (D9G8) Rabbit mAb	3398T	Cell Signaling Technology
Anti Rabbit IgG (H+L) Alexa Fluor 488	2975S	Cell Signaling Technology
Anti Rabbit IgG (H+L) Alexa Fluor 594	8889S	Cell Signaling Technology
Anti Mouse IgG (H+L) Alexa Fluor 488	4408S	Cell Signaling Technology
Anti Mouse IgG (H+L) Alexa Fluor 594	8890S	Cell Signaling Technology
PSD-95 Polyclonal Antibody	516900	Thermo Fisher
Anti-ATF6 Antibody	ab227830	Abcam
Anti-GRP78 BiP Antibody	ab21685	Abcam
PSD-95 (D27E11) XP ® Rabbit mAb	3450T	Cell Signaling Technology
Anti-beta III Tubulin Antibody	ab78078	Abcam
BDNF Polyclonal Antibody	PA5-111802	Thermo Fisher
Anti-VGluT1 Antibody	ab227805	Abcam
